# Beyond the Circulating Renin–Angiotensin Aldosterone System

**DOI:** 10.3389/fendo.2014.00104

**Published:** 2014-07-02

**Authors:** Walmor C. De Mello

**Affiliations:** ^1^School of Medicine, Medical Sciences Campus, University of Puerto Rico, San Juan, PR, USA

**Keywords:** renin–angiotensin systems, editorial, RAAS, pathophysiology, oxidative stress

The activation of the classical renin–angiotensin aldosterone system (RAAS) is known to be involved in the regulation of blood volume and blood pressure and plays an important role in cardiovascular pathology including hypertension and heart failure. Evidence is now available that independently of the classical RAAS, several RAAS components are expressed in cells from different organs including the heart and kidney and are able to change important physiological properties like cell communication, heart excitability, and activation of ionic channels and cell volume when applied locally to the cells (1) or systemically, independently of blood pressure. In cardiac cells, swelling induced by angiotensin II (Ang II), is counteracted by angiotensin (1–7) [Ang (1–7)] with consequent decrease of swelling-dependent chloride current helping the re-establishment of cell volume (2). Recently, it was found that Ang (1–7) re-establishes cell communication impaired by cell swelling in cardiac muscle raising the possibility of a beneficial effect of the hexapeptide during myocardial ischemia (3). These findings have important clinical implications (1, 4) and represent a novel and fruitful pathway to be followed to better understand the role of the RAAS in different pathological conditions. Furthermore, they offer the opportunity for the development of new therapeutic agents.

Although studies performed on transgenic animals generated controversial results, evidence is available that the overexpression of some components of RAAS like Ang II on cardiac muscle, elicit ventricular hypertrophy independently of changes in arterial blood pressure (5). Furthermore, the identification of some of the RAAS components inside the cell including the nucleus and mitochondria (6–8) and the results achieved dialyzing Ang II or renin intracellularly (1, 7), supports the notion that there is an intracellular component with functional properties (the intracrine effect) (1, 7). In arterial myocytes from vascular resistance vessels, for instance, intracellular Ang II has an effect opposite to that of extracellular Ang II on vascular tone (9) suggesting an important intracrine effect of the peptide on peripheral resistance. Furthermore, the (pro) renin receptor (PRR), mainly located intracellularly (10, 11), is a new member of the RAS, originally considered to be involved in the regulation of blood pressure. Recent observations using transgenic animals over-expressing PRR demonstrated that PRR is an accessory protein of V-ATPase that plays an important role in the regulation of several cellular homeostatic processes including autophagy (11).

The harmful effects of Ang II on cardiovascular and renal systems inducing remodeling, seems, in part, related to increase in oxidative stress. The discovery of angiotensin converting enzyme 2 (ACE2) (12) and the evidence that it promotes the formation of Ang (1–7) from Ang II in animal models, represented an important chapter in the studies of RAAS because Ang (1–7) counteracts many effects of Ang II (13) including the enhancement of oxidative stress induced by Ang II. Further studies are, however, necessary to confirm if these beneficial effects of Ang (1–7) are present in humans.

In this Research Topic, the pathophysiological role of local RAAS in different tissues and organs are reviewed by different authors, each one expert in their respective fields (14–18). We hope these articles will help the development of future investigation of this important topic.

## Conflict of Interest Statement

The author declares that the research was conducted in the absence of any commercial or financial relationships that could be construed as a potential conflict of interest.
